# Impedimetric DNA Sensor Based on a Composite of Electrochemically Reduced Graphene Oxide and Polyproflavine Electropolymerized from Natural Deep Eutectic Solvent for Anthracycline Medications Determination

**DOI:** 10.3390/bios15060385

**Published:** 2025-06-14

**Authors:** Anastasia Goida, Tatiana Krasnova, Rezeda Shamagsumova, Vladimir Evtugyn, Anatoly Saveliev, Anna Porfireva

**Affiliations:** 1 A.M. Butlerov’s Chemistry Institute, Kazan Federal University, 18 Kremlevskaya Street, 420008 Kazan, Russia; aigojda@kpfu.ru (A.G.); tankulikova@int.kpfu.ru (T.K.); rezeda.shamagsumova@kpfu.ru (R.S.); 2 Interdisciplinary Center of Analytical Microscopy, Kazan Federal University, 18 Kremlevskaya Street, 420008 Kazan, Russia; vgevtjugin@kpfu.ru; 3 Institute of Ecology, Biotechnology and Nature Management, Kazan Federal University, 18 Kremlevskaya Street, 420008 Kazan, Russia; anatoly.saveliev@kpfu.ru

**Keywords:** proflavine, graphene oxide, nanocomposite, natural deep eutectic solvent, electropolymerization, electrochemical DNA sensor, electrochemical impedance spectroscopy, anthracycline medications, point-of-care diagnostics

## Abstract

A novel nanocomposite based on electrochemically reduced graphene oxide (ERGO) and electropolymerized polyproflavine (PPFL) was obtained within a “one-pot” synthesis from natural deep eutectic solvent (NADES). NADES consisted of citric acid, glucose, and water in a molar ratio of 1:1:6. The synthesis was carried out in potentiostatic mode by consequent potential application in cathodic and anodic areas. The composite was applied to develop the impedimetric DNA sensor for anthracycline determination. The sensor has provided linear range from 10 nM to 0.1 mM for doxorubicin, from 1 pM to 10 nM for epirubicin, and from 10 pM to 10 nM for idarubicin, with the limit of detection 3 nM, 1 pM, and 5 pM, respectively. The concentrations of doxorubicin below 10 nM did not have any other influence on epirubicin and idarubicin determination despite their molecular structure similarity. The sensor developed was used for the determination of anticancer medications, such as doxorubicin, epirubicin, and idarubicin, in their standard solutions, pharmaceuticals, artificial, and human urine samples. It is worth noting that the additions of mannitol and lactose, which are the stabilizers of the pharmaceuticals, exhibited an interfering effect on the sensor response.

## 1. Introduction

Cancer is one of the main causes of death in the world. The anthracycline medications, which have a cytostatic effect, are widely applied for different cancer types, even though they have a high level of cardiovascular toxicity. As a result, after anthracycline intake, the patients can experience some consequences such as heart failure with reduced ejection fraction and pericardial diseases or those connected to the valve, coronary artery, and vascular system [1,2]. Within the medication high concentration intake, there is a risk of cardiotoxicity and other side effects, for example, skin damage, calvity, nausea, vomiting, etc., whereas low concentrations cannot provide a sufficient therapeutic effect. Therefore, the thorough monitoring of anthracycline levels in the body fluids is of great importance [3].

The most conventional methods for anthracycline medication determination are high-pressure liquid chromatography and spectroscopy. Despite their high sensitivity, these methods have several disadvantages, such as expensive instrumentation, the necessity of high-grade reagents, strict requirements for the staff skills, along with the labor- and time-consuming analysis [3]. Electrochemical sensors are advantageous devices that are widely implemented in medical practice for early diagnostics and determination of medicinal preparations or biomarkers [4,5]. Being rather cheap, they are compatible with commercially available equipment and provide a fast and sensitive response. Another incontestable advantage of electrochemical sensors is the opportunity to carry out the analysis of small sample volumes, including biological ones. Also, these sensors can provide the development of single-use devices based on screen-printed electrodes for routine medical analysis.

Biosensors consist of a biological element to capture the analyte and a transducer to convert the captured compound in the sample into a detectable response for qualitative or quantitative detection. These biorecognition elements can be presented by enzymes, whole cells, DNA and RNA, antibodies, or antigens. Different kinds of optical biosensors based on colorimetry or fluorimetry, surface plasmonic biosensors, electrochemical DNA sensors, immunosensors, enzyme sensors, and aptasensors based on voltametric, potentiometric or impedimetric measurements are presented as a promising tool for the rapid and reliable response on the analyte presence and/or its amount in the sample as well as low cost, miniaturization and portability of biosensors [6].

In the last decade, there was an essential trend of using the natural deep eutectic solvents (NADES) for novel materials development [7]. Such solvents have already been used successfully to obtain electropolymerized coatings, electrodeposited metallic and oxide nanostructures, and carbon nanomaterials dispersion [8,9,10]. The resulting materials had different structures and properties compared to their analogues obtained from aqueous and organic media. To obtain deep eutectic solvents (DES), the hydrogen bond donor and acceptor components are mixed. Their molecular interaction leads to the mixture’s melting point decreasing when compared to the values typical for the original components. Nowadays, the best-known DESs are those based on choline chloride. Organic acids and sugar-based NADES are less frequently used, but they are more challenging in view of the potentially possible contents range due to the variety of their origin components [11].

Graphene-based materials have found their wide application in the context of electrochemical sensors and biosensors [5,12]. Electrochemically reduced graphene oxide (ERGO) plays a special role among them [13]. Electrochemical reduction from graphene oxide (GO) towards ERGO is carried out in the solution volume or on the electrode surface at the cathodic potential area. GO can be reduced to the ERGO both with potentiostatic and potentiodynamic modes. The first one implies constant potential application during the definite time interval. The second one suggests the multiple cycling of the potential during the reduction process. Contrary to chemical reduction, the electrochemical mode led to the product formation with no chemical contaminants. Electroreduction potential choice has an influence on GO reduction degree, as well as the content and structure of functional oxygen-containing groups on its surface [14]. Although there are some drawbacks of ERGO, such as structural defects and mass transfer complications, this graphene-like material has some promising advantages, which are high conductivity and good stability of the coating. Also, sufficient scalability can be achieved easily after some optimization stages. One of the most demanded properties of ERGO is the increase of the efficient surface of the working electrode, which leads to signal-to-noise decreasing and better electric conductivity of the support. This can provide more efficient binding of the bioreceptor. Another benefit of ERGO is electrocatalytic activity. ERGO layered nanosheets are the most convenient resulting structures, though depending on working conditions and template presence, there can be nanofoams and nanopores structures obtained [15,16]. As it was shown in literature, the use of graphene-based nanomaterials with more complicated architecture for electrochemical sensors development has improved the electron transfer parameters and their analytical characteristics [17].

Electropolymerized acridine dyes are promising surface modifiers for electrochemical sensors as they have the properties of heterogeneous electron transfer mediators. The representatives of this class of dyes can interact with double-stranded DNA on the intercalation principle [18]. Acridine dyes can be electropolymerized from either aqueous or DES media. Previously, our scientific group has developed a range of electrochemical sensors for anthracycline drugs and DNA damage determination based on polymeric forms of acridine dyes [8,19].

In this work, the possible application of “one-pot” synthesized ERGO and electropolymerized proflavine (PPFL) nanocomposites in a DNA sensor for anthracycline medications determination has been demonstrated for the first time ever. Also, there was a difference shown for ERGO morphology depending on electroreduction from the DES mode. The sensor developed can be found in its application in the area of pharmaceutics and medical diagnostics for the “point-of-care” concept realization.

## 2. Materials and Methods

### 2.1. Reagents

3,6-diaminoacridine hydrochloride (proflavine) (dye content 95%), graphene oxide (GO) 2 mg/mL dispersion in water, doxorubicin hydrochloride, idarubicin hydrochloride (98%), deoxyribonucleic acid (DNA) from salmon sperm, and deoxyribonucleic acid from calf thymus were received from Sigma–Aldrich (Darmstadt, Germany). D-Glucose anhydrous, citric acid monohydrate, and epirubicin hydrochloride (98.2%) from Alfa Aesar (Ward Hill, MA, USA) were used. All reagents were of at least analytical grade. Working solutions were obtained with deionized Millipore^®^ water (Burlington, MA, USA). 0.025 M phosphate buffer (PB) containing 0.1 M KCl, pH 7.0, was used to carry out voltametric and impedimetric measurements; 10% solutions of NaOH or H3PO4 were added to adjust the pH value in appropriate experiments. Doxorubicin-LANS^®^ (“Verofarm”, Volginskiy, Russia), Doxorubicin-TEVA (“Pharmachemie”, B.V., Haarlem, The Netherlands), and idarubicin preparation Zavedos^®^ (Pfizer Inc., New York, NY, USA) used as pharmaceutical samples were provided by the local pharmacies.

### 2.2. Apparatus

Potentiostat-galvanostat AUTOLAB PGSTAT 302N (Metrohm Autolab b.v., Utrecht, The Netherlands) equipped with FRA2 module was used to perform the electrochemical measurements (cyclic voltammetry and EIS) at ambient temperature. The DEC 248 printer (DEK, London, UK) and Lomond PE DS Laser Film (thickness 125 µm, Lomond Trading Ltd., Douglas, Isle of Man, UK) were used for printing the screen-printed carbon electrodes (SPCEs). To obtain three electrode system with the dimensions of 11 mm × 27 mm and geometric area of working electrode equal to 3.8 mm^2^ the conductive tracks of PSP-2 silver paste (Delta–Paste, Moscow, Russia) and carbon/graphite paste C2030519P4 (Gwent group, Pontypool, UK), were covered with insulating layer based on dielectric paste D2140114D5 (Gwent group, Pontypool, UK) and treated at the temperature of 80 °C. All the potentials were given against the Ag pseudo-reference electrode, printed together with other electrodes. The boxed connector for screen-printed electrodes (DropSens, S.L., Asturias Llanera, Spain) was used to provide the registration of electrochemical response. The electrochemical characterization of the nanocomposite modifier was performed with cyclic voltammetry (CV) and electrochemical impedance spectroscopy (EIS) methods.

The conditions of EIS experiments are the following: the potential frequency range was from 100 kHz to 0.04 Hz, and the applied sine potential amplitude was equal to 5 mV. The measurements were carried out in 0.025 M PB in the presence of an equimolar mixture of 0.01 M [Fe(CN)6]3-/4- as a redox probe at the equilibrium potential calculated as a half-sum of the peak potentials recorded. The equivalent circuit R(R1C1)(R2C2) was used for the evaluation of EIS parameters using NOVA 1.11 software (Metrohm Autolab b.v., Utrecht, The Netherlands).

Scanning electron microscopy (SEM) images of the modifying coatings were obtained with Metrohm DropSens DRP-110 screen-printed electrodes as supports (DropSens, S.L., Asturias Llanera, Spain) using the high-resolution field emission scanning electron microscope Merlin™ (Carl Zeiss AG, Oberkochen, Germany) and the ZeissSmartSEM software (V05.06). Elemental mapping data were obtained using the Oxford Instruments X-Max 80 equipment for energy dispersive spectrometry (Oxford Instruments, Oxford, UK) with Aztec 3.1 software.

DES mixture synthesis suggested the stirring of the original components with a vortex (Biosan SIA, Riga, Latvia), followed by ultrasonication in an ultrasonic bath, Wise Clean with 50 Hz frequency (DAIHAN Scientific Co, Ltd., Wonju, Republic of Korea).

Statistical analysis was performed using OriginPro 8.1 software (OriginLab Corp., Northampton, MA, USA).

### 2.3. SPCE Surface Modification

Initial NADES preparation in the absence of acridine dye included mixing of citric acid, D-glucose, and water in a molar ratio of 1:1:6 (0.21 g of citric acid monohydrate, 0.18 g of D-glucose, and 90 µL of deionized water, Millipore Q^®^). 0.085 M proflavine (6.6 mg) was added to the initial NADES to obtain NADES containing the dye. NADES with GO was obtained after adding the water GO dispersion to the citric acid monohydrate and D-glucose, so that the total volume of both GO water dispersion and water was equal to 90 µL. GO content in NADES was varied in the range from 0.2 to 0.6 mg/mL. NADES containing 0.6 mg/mL GO and 0.085 M proflavine were prepared by the addition of 6.6 mg of the acridine dye and 90 µL of 2 mg/mL GO dispersion in water to the appropriate molar ratio of citric acid and D-glucose. To homogenize NADES components, 1 min vortex stirring followed by 30 min ultrasonication was performed as presented in recent investigations of our scientific group [8].

To obtain ERGO coatings, 100 µL of NADES with GO were applied on the SPCE three-electrode surface, and then 10-fold potential scanning was performed in the potential range from 0.05 to −1.5 V at a scan rate of 0.1 V/s. The appropriate coating was denoted as ERGO_NADES_.

In case of layer-by-layer assembling, the dye electropolymerization was carried out on the top of ERGO_NADES_ from 100 µL of NADES with 0.085 M proflavine with 20-fold potential scanning in the range from −1.2 to 1.2 V at a scan rate of 0.1 V/s.

When applying NADES containing 0.6 mg/mL GO and 0.085 M proflavine simultaneously, three modes of electropolymerization were used, such as potentiodynamic, potentiostatic, and mixed ones. In potentiodynamic mode, the potential was cycled 20 times between 1.2 and −1.5 V at a scan rate of 0.1 V/s. In potentiostatic mode, the potential was first applied for 300 s at −1.5 V and then for 300 s at 1.2 V in the same NADES aliquot. For mixed-mode implementation, the two-step potential application was utilized. At the first step, −1.5 V was applied for 300 s, at the second step, 1.2 V was applied for 300 s with further 20-fold cycling in the potential range from −1.2 to 1.2 V at a scan rate of 0.1 V/s. The appropriate coating was denoted as ERGO-PPFL_NADES_.

After the nanocomposite development, the modified electrodes were rinsed with deionized water, followed by drying at ambient temperature. To avoid the presence of unbound layer components, the electrochemical stabilization of the polymer coating was performed prior to the DNA immobilization. The stabilization protocol suggested the 10-fold multiple potential scanning between −0.6 and 0.6 V at a scan rate of 0.1 V/s in 100 µL of PB.

The application of a 2 µL aliquot of 1 mg/mL aqueous solution of DNA onto the SPCE modified with ERGO-PPFL_NADES_ provided the immobilization of DNA from salmon sperm (DNAss) or DNA from calf thymus (DNAct). The complete drying or 5, 10, or 20 min incubation was used as the immobilization modes, followed by deionized water washing to remove the unbound biomolecules.

### 2.4. Anthracycline Medication Determination in Standard Solutions and Real Samples

The SPCE modified with ERGO-PPFL_NADES_/DNAss was incubated for 20 min in 2 µL of doxorubicin (10 nM–0.1 mM), epirubicin (1 pM–1 µM), or idarubicin (1 pM–0.1 µM) aqueous solutions. Subsequently, the electrode was washed with deionized water and dried in the air at ambient temperature. Then, providing the hydrolytic contact of the three-electrode system, 100 µL of PB with a 0.01 M equimolar mixture of the [Fe(CN)_6_]^3−/4−^ redox probe was drop-cast onto the electrode surface, and the EIS measurements were carried out.

Doxorubicin-LANS^®^, Doxorubicin-TEVA^®^, и Zavedos^®^ medications were dissolved in 0.9% NaCl solution with subsequent dilution to the working concentrations.

The artificial urine consisted of 20 mM KCl, 49 mM NaCl, 15 mM KH_2_PO_4_, 10 mM CaCl_2_, 18 mM NH_4_Cl, and 18 mM urea [20]. Human urine was provided by a healthy donor with a sample pH value adjusted to 7.0. The precipitate obtained during the pH value correction was removed by filtration on filter paper.

To assess the stability of the sensors developed, there were several experiments with the set of modified electrodes. Each of the sets included 15 electrodes based on the same reagents and used once in a three-day period. After two weeks, the sensor response decreased by more than 40%, which was regarded as the sensor to be invalid.

To estimate the recovery values, the ratio was calculated for the sensor response registered in a real sample or in the presence of interfering compounds to that of the standard solution in the working buffer. The result of such a calculation was presented in %.

## 3. Results

### 3.1. Electrochemically Reduced Graphene Oxide Production

**Figure 1:**
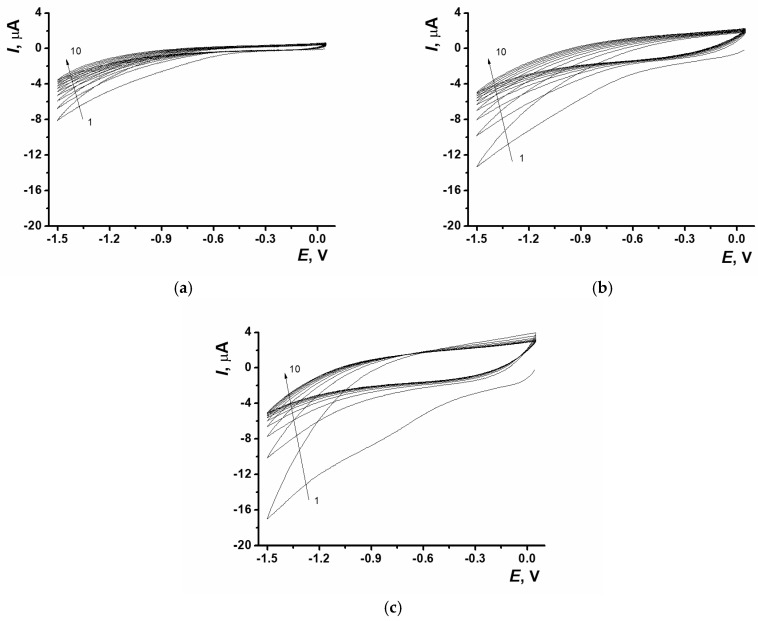
Cyclic voltammograms of ERGO obtained with different content of GO in NADES; (**a**) 0.2 mg/mL, (**b**) 0.33 mg/mL, (**c**) 0.6 mg/mL; SPCE, 0.05…−1.5 V, 0.1 V/s, 10 cycles. The arrows indicate the number of cycles.

Stable, homogeneous, transparent dispersions were obtained when GO was dispersed in NADES with an ultrasonic bath. This allowed tuning the degree of nanomaterial dispersion in NADES (Figure S1 in Supplementary Materials). GO was electrochemically reduced in potentiodynamic mode. For this purpose, the dispersion was drop-cast onto the SPCE surface with the following 10-fold potential scanning. The distinctive signal of GO electrochemical reduction was recorded at the cathodic curve of voltammogram at −1.5 V. At the first potential scan the cathodic current value depended on the GO concentration in dispersion and was equal to 7.8 ± 1.9, 12.5 ± 2.5, 16.3 ± 2.3 µA (n = 6) for 0.2, 0.33, 0.6 mg/mL GO in NADES, relatively (Figure 1).

**Figure 2:**
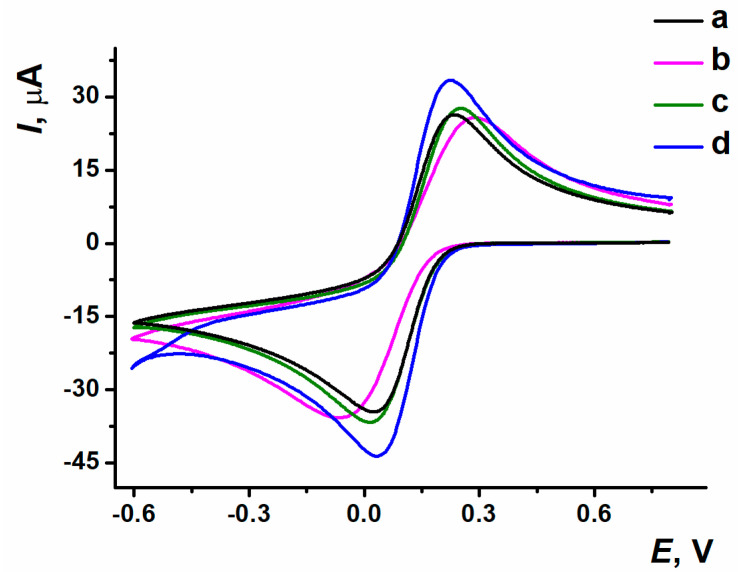
Cyclic voltammograms recorded on (a) bare SPCE; SPCE/ERGO with GO dispersion concentrations: (b) 0.2 mg/mL, (c) 0.33 mg/mL, (d) 0.6 mg/mL; 0.025 M PB, pH 7.0, 0.01 M K_3_[Fe(CN)_6_], 0.8…−0.6 V, 0.1 V/s.

The response of 0.01 M K_3_[Fe(CN)_6_] as a diffusional free redox probe was used for the optimization of GO loading onto the electrode. The cyclic voltammograms obtained are shown in Figure 2. The highest redox peak values and the minimal potential difference were achieved at the ERGO coating synthesized from GO with a 0.6 mg/mL concentration, so this GO content has been chosen for the following experiments with dye involvement.

### 3.2. Electrochemical Polymerization of Proflavine and the Estimation of ERGO-PPFL_NADES_ Composite Electrochemical Characteristics

**Figure 3:**
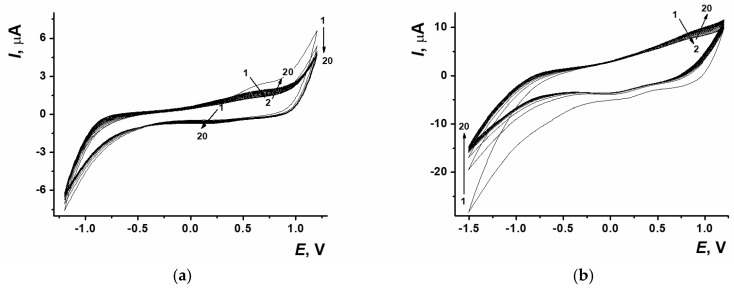
(**a**) Cyclic voltammograms of proflavine electropolymerization on SPCE/ERGO_NADES_, −1.2…1.2 V, 0.1 V/s, 20 cycles. (**b**) Cyclic voltammograms of ERGO-PPFL_NADES_ composite, 1.2…−1.5 V, 0.1 V/s, 20 cycles. The arrows indicate the number of cycles.

There were several approaches used to obtain the coatings with electropolymerized proflavine. In the first case, layer-by-layer modification of the sensor surface was used, so the dye electropolymerization from DES was carried out onto ERGO_NADES_ (Figure 3a). The second approach implied the electropolymerization of proflavine being performed simultaneously with electrochemical reduction of GO towards ERGO from the DES containing 0.6 mg/mL GO and 0.085 M proflavine (Figure 3b). Both methods are based on the potentiodynamic electropolymerization mode.

The location and morphology of the peaks recorded on cyclic voltammograms of proflavine polymerization on SPCE/ERGO_NADES_ were similar to those obtained earlier on the bare SPCE surface [7]. In case of coating including both of the components, there was a signal at −1.5 V on the cathodic curve, which corresponded to GO reduction. Both GO reduction and proflavine electropolymerization currents had higher values, which indicated the amplification of PPFL mediator properties and ERGO electroconductivity influence due to their joint presence.

For the assessment of the opportunity of the coatings obtained application in sensors, the estimation of the PPFL redox current value and stability is required. The stabilization of polymer coatings developed in different electropolymerization modes was performed by multiple potential scans in 100 µL of PB. One-step electropolymerization provided better electrochemical characteristics of the resulting coatings when compared with layer-by-layer deposition (Figure S2), so this approach has been chosen for the following experiments.

Along with the potentiodynamic mode of electropolymerization, the potentiometric and mixed modes have been tested (Figure S3). It is known that the electropolymerization in potentiodynamic mode results in the formation of a thin polymeric coating with satisfactory adhesion. As for the potentiostatic mode, it leads to a larger quantity of polymer being deposited but being wasted with side products [21].

**Figure 4:**
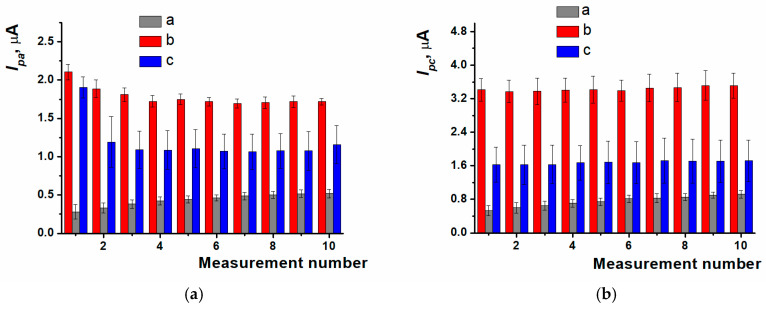
Response stability of (**a**) oxidation current values, (**b**) reduction current values on ERGO-PPFL_NADES_ coatings obtained with (a) potentiodynamic; (b) potentiostatic; (c) mixed modes; 0.025 M PB, pH 7.0, −0.6…0.6 V, 0.1 V/s.

The response stabilization was carried out within a 10-fold potential scanning in working buffer solution for polymer coating obtained with different modes of electropolymerization (Figure 4). For PPFL coating developed in potentiodynamic mode, the stabilization resulted in the efficient treatment of the surface, which was indicated by an increase in peak currents. During the stabilization of the coating obtained with the potentiostatic mode, there was the removal of both the dye monomer entrapped and the reaction side products, which was expressed as the oxidation peak current decreasing in the first cycles of scanning. When using the mixed mode, a dramatic decrease in oxidation peak current occurred between the first cycle and the second one. The following potential scanning resulted in irregular changes of redox peak currents, which led to the response reproducibility worsening.

**Figure 5:**
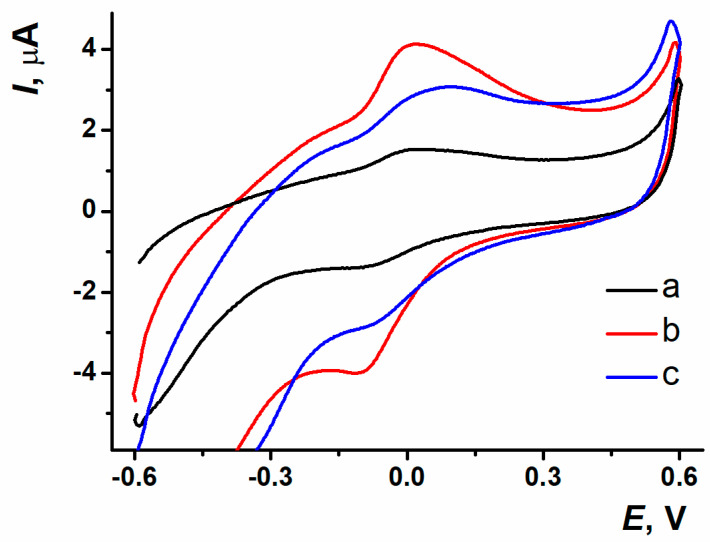
Voltametric response of PPFL obtained with (a) potentiodynamic; (b) potentiostatic; (c) mixed modes; 0.025 M PB, pH 7.0, −0.6…0.6 V, 0.1 V/s.

The sensors on the base of SPCE/ERGO-PPFL_NADES_ obtained with different electropolymerization modes were placed into the working buffer solution. The PPFL peaks were distinctive because of current values and morphology depending on the electropolymerization mode (Figure 5).

The reproducibility and resolution of polymeric coating redox peaks were better for the electroactive polymer obtained with the potentiostatic mode of electropolymerization. This was the reason for this mode to be chosen as the most efficient approach to the modification of the surface layer.

**Figure 6:**
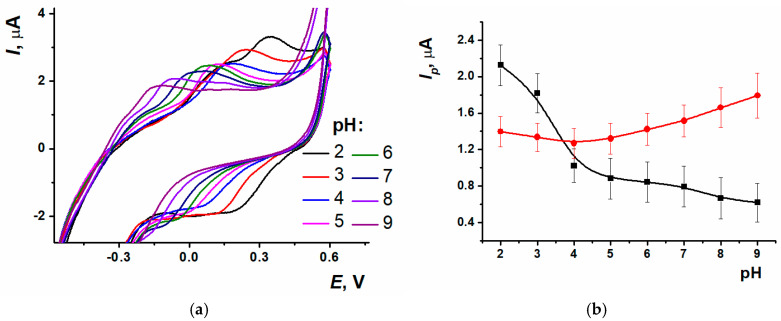
(**a**) Cyclic voltammograms of SPCE/ERGO-PPFL_NADES_ at different pH values, (**b**) Oxidation (black) and reduction (red) peak currents dependence on pH; 0.025 M PB, n = 9.

Electrochemical characteristics were established for the sensor based on SPCE/ERGO-PPFL_NADES_. Varying the pH value from 2.0 to 9.0 led to peaks of polymeric form shifting towards the cathodic potentials (Figure 6a). The response recorded for the reduction peak current at acidic pH values was quite stable, whereas the peak current increased in the range from 4.0 to 9.0. Oxidation peak current decreased in the 2.0–5.0 range; however, from 5.0 to 9.0 it was changed insignificantly (Figure 6b).

For the equilibrium potential *E_m_* calculation, a half-sum of the anodic and cathodic peak potentials of PPFL was used. The pH dependency was linear in the range from 2.0 to 9.0 with a slope value of −0.061 ± 0.002 V/pH. The slope value was close to the theoretical Nernstian one, indicating the equal number of electrons and protons transferred in the electrode reaction.

Figure S4 shows the cyclic voltammograms recorded for SPCE/ERGO-PPFL_NADES_ at different scan rates. The slope of bilogarithmic dependency of peak current (*Ip*) on the potential scan rate (*ν*) for the SPCE/ERGO-PPFL_NADES_ demonstrates the significant contribution of adsorption processes to the limiting step of polymer redox conversions (d(log*I_pa_*)/d(log*ν*) = 0.867 ± 0.009 and d(log*I_pc_*)/d(log*ν*) = 0.927 ± 0.012, relatively).

### 3.3. ERGO_NADES_ and ERGO-PPFL_NADES_ Coatings Characterization with SEM Images

**Figure 7:**
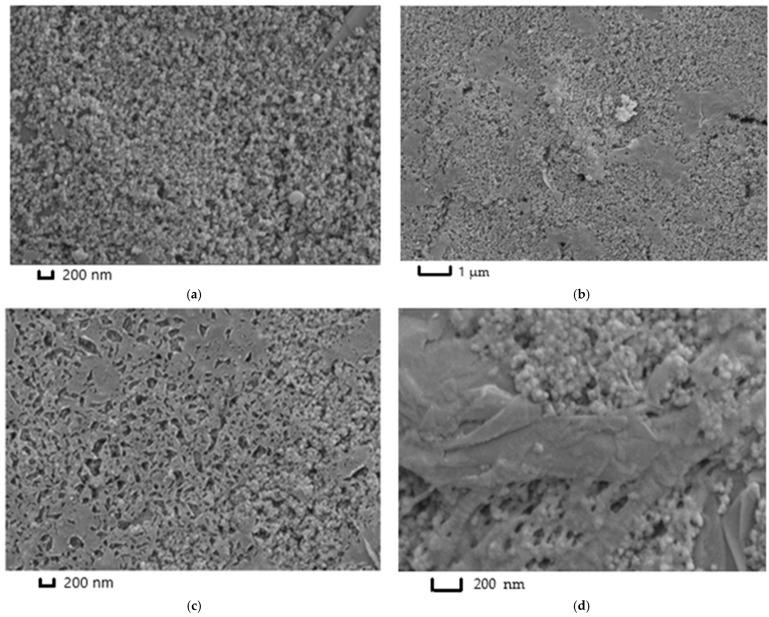
SEM microimages of (**a**) bare SPCE; (**b**,**c**) ERGO_NADES_ at different magnifications; (**d**) ERGO-PPFL_NADES._

The microimages obtained with SEM have demonstrated the differences in ERGO morphology, which depended on the mode of GO electrochemical reduction from NADES (Figure 7). Bare SPCE has exhibited the microgranulated coating formed by the carbon ink particles, which were used for the electrode printing (Figure 7a). In case of the coating of ERGO_NADES_ obtained with multiple scanning mode in the absence of the dye, single sheets of ERGO were observed at low magnifications. These sheets were smoothly distributed on the surface of carbon nanomaterial (Figure 7b). It was found that these ERGO sheets had a lot of shattering at high magnifications (Figure 7c). One-step synthesis of the ERGO-PPFL_NADES_ composite with potentiostatic mode led to the formation of a conventional dense multilayered structure of ERGO (in the middle of Figure 7d) on which the sites of polymer growth were localized, filling the pores of carbon material with the following dense polymeric layer formation.

**Figure 8:**
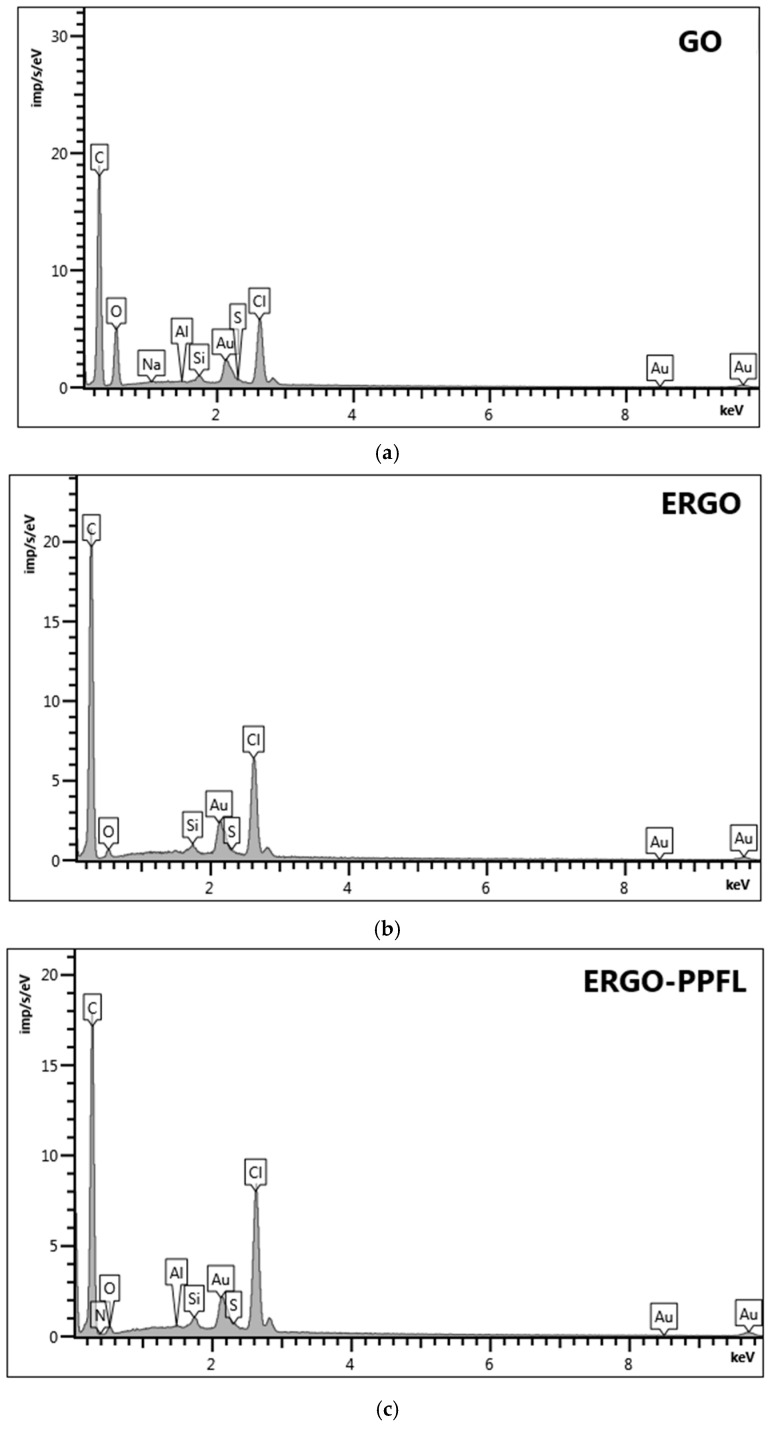
Elemental mapping data of (**a**) SPCE/GO, (**b**) SPCE/ERGO_NADES_, (**c**) SPCE/ERGO-PPFL_NADES_.

The following results of the element mapping data for the GO, ERGO_NADES_, ERGO-PPFL_NADES_ composite are presented in Figure 8a–c and Table S1a–c.

Metrohm DropSens DRP-110 screen-printed electrodes were used as a support (DropSens, S.L., Asturias Llanera, Spain) for the elemental mapping data analysis. They consist of ceramic support with carbon-based ink, which provides a high initial level of carbon content. Further modification of the electrode with GO suspension in the water led to a carbon:oxygen percentage ratio close to 3:1 (71.03 and 24.57 weight %, respectively). The GO reduction to ERGO_NADES_ changed this ratio significantly to 24:1 (90.72 and 3.79 weight% %, respectively), indicating the successful reduction of the oxygen-containing groups at the graphene-based surface. Electrochemical treating of ERGO_NADES_ in the buffer solution containing chloride ions indicated their inclusion into the surface layer (5.07 weight %). “One-pot” synthesis led to the formation of the nanocomposite ERGO-PPFL_NADES_, resulting in the appearance of the N element response being rather low in comparison with the total amount of carbon. Another confirmation of PPFL inclusion into the modifying layer was the rise of chloride-ion content (from 5.07 to 6.35 weight %), demonstrating their successful participation in the polymer doping mechanism.

### 3.4. DNA Sensor Assembling

**Figure 9:**
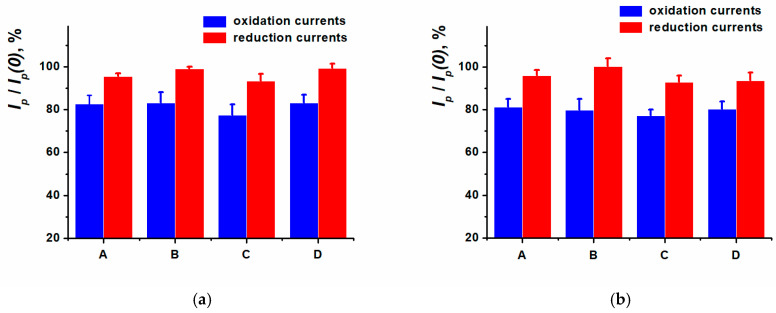
Relative peak current changes (before (*Ip*(*0*)) and after (*Ip*) DNA incubation). Drying (A) and incubation for 5, 10, 20 min (B, C, D) in an aliquot of (**a**) DNAct, (**b**) DNAss. Cyclic voltammetry, 0.025 M PB, pH 7.0, −0.6…0.6 V, 0.1 V/s.

Double-stranded DNAss or DNAct was physically adsorbed onto the SPCE/ERGO-PPFL_NADES_ for DNA sensor development. The DNA solution aliquot was dried at ambient conditions or incubated for 5, 10, or 20 min in the Eppendorf tube to avoid evaporation. The relative changes of PPFL redox peaks are shown in Figure 9.

The inclusion of DNAct or DNAss non-conductive molecules into the surface layer resulted in the electron transfer hindrance and insignificantly decreased the currents in the cyclic voltammograms recorded. The oxidation peak current influenced by DNA has changed more prominently compared to the reduction peak current. As a result, 10 min incubation of SPCE modified with ERGO-PPFL_NADES_ in DNAss or DNAct solution was chosen as a working method of DNA sensor assembly for anthracycline medications determination.

### 3.5. Anthracycline Medications Determination

The DNA sensor based on the composite ERGO-PPFL_NADES_ was developed for anthracycline group cytostatic medications (doxorubicin, epirubicin, idarubicin) determination. The anthracyclines are well-known compounds playing the role of intercalators; their pharmaceutical effect occurs due to the interaction with the DNA molecule as a biological target. When the DNA interacts with such medication, the incorporation of planar tetracyclic chromophores occurs between the neighboring pairs of nucleic bases. Stabilization of the complex formed takes place due to electrostatic interactions between phosphate groups of the DNA backbone and positively charged amino groups of sugar fragments. The removal or replacement of the amino glucosamine residue leads to a critical decrease in medication binding constants. The medication binding with the DNA molecule can take place along the DNA grooves. For example, there is an opportunity shown for the hybrid mechanism of doxorubicin interaction, when both the intercalation and binding along the DNA grooves are possible [22]. As for calf thymus DNA-doxorubicin complex, the opportunity of two different complexes (PD1 and PD2) formation was shown depending on the concentrations of doxorubicin and DNA. PD1 was formed if C(DOX)/C(DNA) < 0.3, whereas PD2 occurred when the anthracycline content exceeded. This complex behavior has also been investigated with model oligonucleotides [poly(dA-dT)]_2_ and [poly(dG-dC)]_2_. PD1 complex formation was classified kinetically as a two-step process in which a fast stage was binding with the groove at the areas of AT nucleotides, and the slow one was the intercalation process at the areas rich with GC nucleotides. The fast stage implied the sugar residue interaction with DNA backbone phosphate groups and the following formation of hydrogen bonds between the OH group of the sugar fragment and oxygen or nitrogen atoms in nucleic bases. This explains a high value of the binding constant obtained for the PD1 complex (K_1_ = 2.3 × 10^8^ M^−1^) compared to the constant for the outside aggregated complex (K_2_ = 9.3 × 10^5^ M^−1^) [23]. In the work [24], different mechanisms of interaction were shown to occur depending on the doxorubicin concentration. At the low concentration of medication, the DNA groove binding and the surface accumulation prevailed, whereas there was an inversion of signal, indicating the mechanism substitution for the intercalation at the high concentration values. Epirubicin interacts with DNA at the areas rich with GC bases, whereby the structural and conformational effects occur, which are connected to the intercalation and outer interaction along the DNA phosphate groups [25]. Epirubicin binding constant for salmon sperm DNA was equal to 3.8 × 10^5^ M^−1^ [26]. FTIR results estimation allowed suggesting that idarubicin interacts with DNA along its major groove through G and C bases. The outer interaction of idarubicin with the DNA strand takes place on its phosphate backbone. The fact of the intercalation was confirmed with UV-vis spectroscopy. The binding constant calculated to be 2.1 × 10^4^ M^−1^ has confirmed the limited binding of idarubicin with DNA [27].

The DNA-medication complex formation has paramount importance for anticancer activity exhibition, but the suppression of DNA-specific functions providing their therapeutic effect should also be noted. Anthracycline medications inhibit topoisomerase II due to the formation of a tertiary complex of DNA-medication-enzyme. The anthracycline molecule is placed on the surface between the active center of the enzyme and DNA segregation sites, therefore, preventing the DNA re-ligation. Moreover, the anthracyclines’ effect mechanism can involve the generation of highly active free radicals and membrane interactions [3,22].

**Figure 10:**
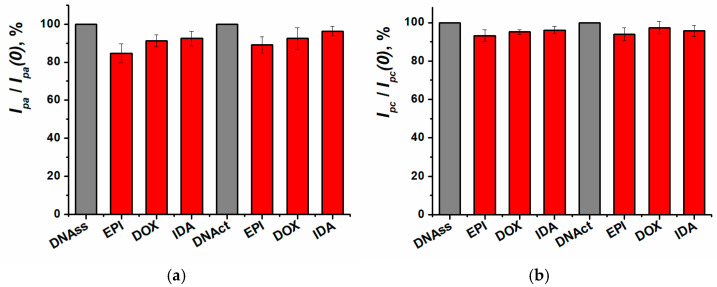
Relative changes of peak currents for (**a**) oxidation, (**b**) reduction on SPCE/ERGO-PPFL_NADES_ (before (*Ip*(*0*)) and after (*Ip*) incubation in 100 nM anthracycline). EPI—epirubicin, DOX—doxorubicin, IDA—idarubicin.

The working conditions for recording biospecific interactions between DNA and anthracyclines were defined using the cyclic voltammetry method. The influence of anthracycline antibiotics intercalation into the DNA molecule on the sensor response was estimated both for low and high-molecular-weight DNA (Figure 10).

DNA source had no influence on the sensitivity of anthracycline antibiotics determination using cyclic voltammetry. For the following experiments, the low molecular weight DNAss was chosen. As DNA sensor incubation in the medication solution did not lead to any significant changes in the cyclic voltammetry response, further investigations of biospecific interactions were carried out with the EIS method. It was previously shown that the impedimetric sensors exhibited greater sensitivity towards the anthracyclines compared to the voltametric approaches [28].

**Figure 11:**
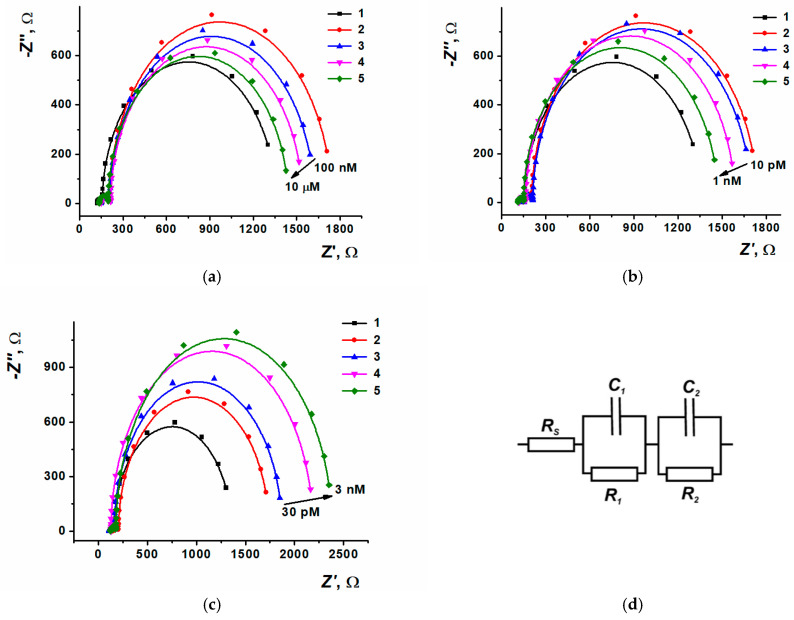
Nyquist diagrams for (**a**) doxorubicin, (**b**) epirubicin, (**c**) idarubicin. Layer content: 1—SPCE/ERGO-PPFL_NADES_, 2—SPCE/ERGO-PPFL_NADES_/DNAss, (3a)–(5a) SPCE/ERGO-PPFL_NADES_/DNAss/DOX (100 nM, 1 µM, 10 µM), (3b)–(5b) SPCE/ERGO-PPFL_NADES_/DNAss/EPI (10 pM, 100 pM, 1 nM), (3c)–(5c) SPCE/ERGO-PPFL_NADES_/DNAss/IDA (30 pM, 300 pM, 3 nM), (**d**) Equivalent circuit used for the EIS parameters evaluation.

The anthracycline incorporation into the DNA strand influenced the biopolymer volume and charge distribution, which has changed the EIS parameters such as capacity and charge transfer resistance of the surface layer. It has also changed the layer permeability for low molecular redox probe (equimolar mixture of 0.01 M [Fe(CN)_6_]^3−/4−^ was used). The charge transfer resistance played a role in the analytical response. Typical Nyquist diagrams obtained both during the DNA sensor assembly and after its incubation in medication solutions are shown in Figure 11; the potentials of the diagrams recording are demonstrated in Table S2. Average values and standard errors for −Z″ and Z′ are presented in Figure S6.

The Nyquist diagram consisted of two semi-circles corresponding to electron transfer in the limiting step of electron exchange at the inner and outer interfaces of the modifying coating. For electrochemical impedance parameters calculation, the equivalent cell scheme *R*(*R*_1_*C*_1_)(*R*_2_*C*_2_) was used, which allowed the interpretation of the measurement results at the modifying layer-solution and electrode-modifying layer interfaces. The appropriate changes of charge transfer resistance (*R*_1_) and surface layer capacity (*C*_1_) depending on the anthracycline concentrations are shown in Figure S5.

Including DNA into the surface layer content enhances the charge transfer resistance as DNA is an insulating component having a negative charge, which leads to additional electrostatic repulsion of negatively charged redox probe molecules. The difference between the responses recorded after the contact with anthracyclines can be related to the realization of different mechanisms of DNA-medication interaction, or the distinct hydrophilic-hydrophobic balance of analytes, or different ability of medications to shield the DNA phosphate backbone charge. Both electrostatic accumulation of anthracyclines in DNA strand grooves and intercalation, with the following interaction of amino sugar with phosphate backbone, can result in the shielding of DNA negative charge. This is expressed by the charge transfer resistance decrease recorded by EIS. The intercalation enhances the DNA strand volume, increasing the sensor surface layer thickness and decreasing its permeability for the redox probe. In this case, the charge transfer resistance after the intercalation will grow. For idarubicin molecules with their better lipophilicity, the changes observed were connected to DNA strand volume increasing, whereas for more polar molecules of doxorubicin and epirubicin, the changes were attributed to the DNA phosphate backbone negative charge neutralization with positively charged aminated ribose residues.

Charge transfer resistance has decreased regularly after the sensor based on SPCE/ERGO-PPFL_NADES_/DNAss was incubated in doxorubicin solution. Doxorubicin concentration linear range based on charge transfer resistance measurement was between 10 nM to 0.1 mM with a limit of detection 3 nM (S/N = 3, Figure S5a). The appropriate equation is presented in Equation (1). The dependency of charge transfer resistance for epirubicin was more complicated, as within low concentrations (from 1 pM to 10 nM), the signal linearly decreased and then stabilized owing to DNA binding sites saturation. Contrary to [22], there was no V-shaped curve, which indicated the replacement of the mechanism from electrostatic accumulation along the grooves towards the intercalation. This allowed us to suggest that the binding of epirubicin and doxorubicin was performed by the intercalation mechanism within efficient shielding of DNA phosphate groups, charged with amino sugar fragments. The linear concentration range of epirubicin was from 1 pM to 10 nM, with a limit of detection of 1 pM (S/N = 3, Figure S5c). The appropriate equation is presented in Equation (2). The charge transfer resistance for idarubicin has linearly increased with the medication concentration growth. The linear range was from 10 pM to 10 nM, with a limit of detection of 5 pM (S/N = 3, Figure S5e). The appropriate equation can be found in Equation (3). The distinguishing effects of intercalation are connected with the different structure of substituents and the polarity of anthracycline molecules.

The analytical characteristics of medication determination are the following:

*R*_1_(DOX), kΩ = (0.648 ± 0.014) + (−0.114 ± 0.002) × lg*c* [DOX, M], R^2^ = 0.997, n = 6 (1)

*R*_1_(EPI), kΩ = (0.504 ± 0.030) + (−0.091 ± 0.003) × lg*c* [EPI, M], R^2^ = 0.9956, n = 6 (2)

*R*_1_(IDA), kΩ = (4.460 ± 0.075) + (0.255 ± 0.008) × lg*c* [IDA, M], R^2^ = 0.9956, n = 6 (3)

Contrary to charge transfer resistance, the changes of surface layer capacity (Figure S5b,d,f) were observed only within the DNA saturation with doxorubicin in the concentration range from 1.0 to 100 µM. The capacity value has increased from 340 to 466 µF (Figure S5b). This is another confirmation of the significant influence of charge control because this suggestion demonstrates the increase of charge carrier number in the sensor surface layer.

The comparison between the sensors presented in the literature and the developed ones is summarized in Table 1. As shown, the use of nanocomposites provides better electrochemical characteristics of the modifier and a more sensitive analytical response.

### 3.6. Impedimetric Determination of Anthracycline Medications in Pharmaceutical Forms and Real Sample Analysis

The reproducibility of anthracycline medication determination was estimated using six sensors based on the same set of reagents. The relative standard deviation (R.S.D.) of charge transfer resistance was 5.0% for doxorubicin, 4.8% for epirubicin, and 8.8% for idarubicin determination. The sensor had a week’s life duration when stored in dry conditions in the fridge (4 °C). Each sensor was used once for avoiding analyte deposition on its surface and the determination results deviation.

**Figure 12:**
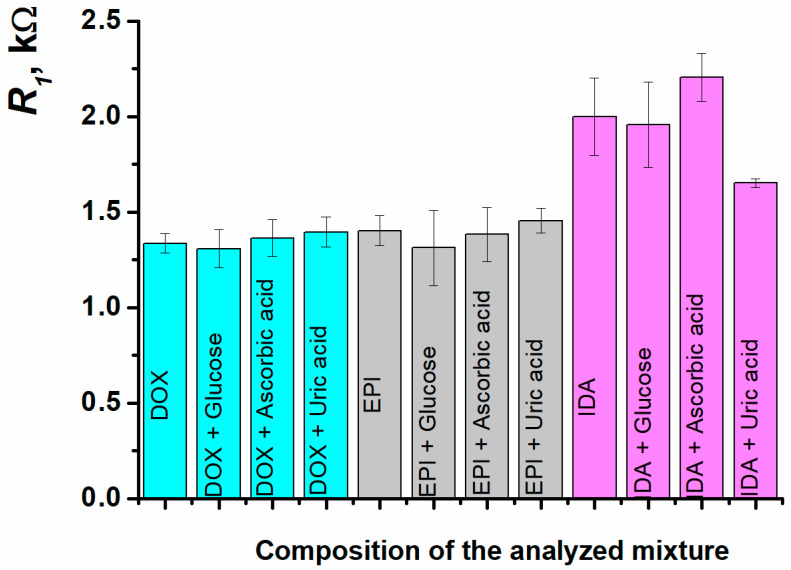
The charge transfer resistance at SPCE/ERGO-PPFLNADES/DNAss for 1 µM doxorubicin, 0.1 nM epirubicin, and 0.3 nM idarubicin determination in the presence of a 10-fold excess of glucose, ascorbic, and uric acids.

The sensor developed was tested for 1 µM doxorubicin, 0.1 nM epirubicin, and 0.3 nM idarubicin determination in the presence of a 10-fold excess of compounds commonly present in the biological fluids, such as glucose, ascorbic, and uric acids. The sensor has demonstrated excellent selectivity for doxorubicin and epirubicin responses towards the interfering components. The response deviation was not more than 6% from the initial value recorded for the standard anthracycline solution. In case of idarubicin, the interfering effects were more evident (2–17%), although the values were still close to the reliable response (Figure 12, Table 2).

The sensor SPCE/ERGO-PPFL_NADES_/DNAss was tested for cytostatic doxorubicin (1 µM) and idarubicin (0.3 nM) determination in commercial medications (Doxorubicin-LANS^®^, Doxorubicin-TEVA^®^, и Zavedos^®^). The recovery obtained is shown in Table 3.

As lactose plays a role as a stabilizer in Zavedos^®^ medication, it had an interfering influence and decreased the idarubicin determination recovery. This effect of lactose was confirmed by the measurements of standard idarubicin solution response with lactose addition.

The recovery values were significantly higher for doxorubicin determination in Doxorubicin-LANS medication than in the presence of Doxorubicin-TEVA. It is supposed to be related to mannitol addition as a stabilizer of this pharmaceutical form. The experiments were performed in the presence of mannitol concentration, which correlated with the content of medication, and they showed a recovery increase.

To evaluate both matrix effect and the opportunity of the sensor developed application for anthracycline medications determination in biological samples, a model sample of artificial urine and a real sample of human urine from a hypothetically healthy donor were used. The analysis of model and real biological samples was carried out with the electrochemical impedance spectroscopy method (Table 4), which showed satisfactory results for doxorubicin, idarubicin, and epirubicin determination. The solution of artificial and human urine samples needed to be diluted with water in a ratio of 1:1 to avoid matrix effects when doxorubicin and epirubicin were determined. Both artificial and human urine constituents did not have any interfering effect on the sensor response within idarubicin determination.

After the working conditions optimization, the recovery value became sufficient, being in the range of 93–107%. Simple procedure of the sample preparation and the rapid response rapidity allows the use of the sensors developed for either medication dosage evaluation in biological fluids of patients within the chemotherapy or treatment plan correction aimed at achieving the best results.

## 4. Discussion

In this work, the polymeric form of proflavine has been used in a nanocomposite with ERGO obtained from DES on the basis of citric acid, glucose, and water. “One-pot” synthesis allowed for simplifying the protocol of the coating assembly and reducing the modification time. The consequent application of the appropriate potentials with potentiostatic mode led to nanocomposite formation, and so it was chosen as a working procedure. Also, the difference between the morphology of ERGO electrochemically reduced from DES with potentiodynamic and potentiostatic modes was shown. In the first case, the porous coatings with lots of shatterings in layered ERGO structure were formed, whereas in the case of potentiostatic mode, there were conventional folded structures on which the following PPFL deposition was performed. DNA sensor response did not depend on biopolymer molecular weight. Its sensitivity for the voltametric mode was insufficient for cytostatic drugs determination. Electrochemical impedance spectroscopy has provided the successful determination of doxorubicin, epirubicin, and idarubicin within a subnanomolar concentration range. The difference in the determination of sensitivity of these medications was related to different polarities and peculiarities of hydrophobic-hydrophilic interactions. The medication’s interaction with DNA took place due to the intercalation mechanism. The negative charge of the DNA phosphate backbone was compensated by the protonated amino groups of sugar residues, with positive charge. The artificial and human urine samples with the working buffer solution in a 1:1 ratio were sufficient to avoid the interfering effect. However, there was the interfering effect of stabilizers on the sensor response towards doxorubicin and idarubicin in pharmaceutical forms. It should be noted within the quantitative analysis performance. Toxic solvents refusal and significant waste volumes decrease within DNA sensor production and operation correspond to “green chemistry” principles. Real samples analysis provides opportunities for the sensor application, both for novel anticancer medications screening and anthracycline antibiotic residues determination in biological fluids aimed at prescribed dose adjustment and chemotherapy efficiency evaluation.

## Figures and Tables

**Table 1:** Comparison of the sensors for doxorubicin, epirubicin, and idarubicin determination presented in the literature.

Analyte	Sensor Content	Method	Concentration Range, LOD	Selectivity Assessment	Real Samples	Refs
DOX	SPCE/ZnO/ MWCNT	DPV	0.007–150.0 µM, 0.002 µM	in 200-folds of K^+^, Na^+^, Mg^2+^, Ca^2+^, NH_4_^+^, Cl^−^, CO_3_^2−^, SO_4_^2−^	doxorubicin injection solution	[29]
	GCE/GO/MoS_2_/ Pd_(4)_-Au_(3_	DPV	0.11–127.5 μM, 0.042 μM	in 100-folds of Ni^2+^, Zn^2+^, Mg^2+^, Cl^−^, NO_3_^−^; 10-folds of Ca^2+^, Cu^2+^, glucose, uric acid, dopamine and ascorbic acid	human urine samples	[30]
	GCE/ mSiO_2_@ MWCNT	DPV	30–750 μM, 14 nM	in 3-folds of ascorbic acid, uric acid, acetaminophen, sulfamethoxazole, glucose, mannose, lactose	human urine samples	[31]
	GCE/poly(Toluidine Blue	DPV	17 nM–8.6 μM, 17 nM	acetate, serine	treated whole plasma, untreated whole plasma, various cell lysates, plasma samples of patients	[32]
	SPCE/ERGO-PPFL_NADES_	EIS	10 nM–0.1 mM, 3 nM	in 10-folds of glucose, ascorbic acid, uric acid	artificial urine, human urine samples, medical preparations: Doxorubicin-TEVA^®^, Doxorubicin-LANS^®^	[This work]
IDA	GCE/SWCNTs/Pt-Pd-ZnO/ds-DNA	DPV	1 nM–65 μM, 0.8 nM	in 1000-folds of K^+^, Na^+^, Li^+^, F^−^, Ca^2+^, 800-folds of alanine and glycine, valine, 500-folds of glucose, 4-folds of doxorubicin and daunorubicin	human urine samples	[33]
	CPE/ MOF-199/PC-rGO	DPV	5–1000 nM, 1.5 nM	in 500-fold Mg^2+^, Na^+^, Ca^2+^, K^+^, Cl^−^, Li^+^, Al^3+^, NH^4+^, dopamine and ascorbic acid	human urine samples, serum samples	[34]
	GPE/MWCNT	DPV	1–1000 nM, 0.2 nM	in 700-fold Mg^2+^, Li^+^, Al^3+^, Na^+^, K^+^, NH^4+^, and dopamine	human urine samples, serum samples	[35]
	GCE/PNR/ TKA/ NR/DNA	DPV, EIS	1 nM–100 μM, 1 nM	in binary solutions of 1000-fold excess of anthracyclines or sulfonylamide preparations (sulfomethoxazole, sulfadiazine, sulfamethazine, and sulfaguanine)	medical preparations: Doxorubicin- LANS^®^ and “Rastocin”^®^	[36]
	SPCE/ERGO-PPFL_NADES_	EIS	10 pM–10 nM, 5 pM	in 10-folds of glucose, ascorbic acid, uric acid	artificial urine, human urine samples, medical preparation Idarubicin-Zavedos^®^	[This work]
EPI	CPE/DES/Pt-SWCNT	DPV	0.001–500 μM, 0.8 nM	in 1000-folds of Li^+^, K^+^, Cl^−^, Br^−^, Mg^2+^, 600-folds of methionine, tryptophan, phenylalanine, valine, 100-folds of ascorbic acid and vitamin B_6_	epirubicin injection solution and dextrose saline samples	[37]
	PGE/ds-DNA/ds-DNA/PP	DPV	0.004–55.0 μM, 1.0 nM	in 1000-folds of K^+^, Cl^−^, Na^+^, Br^−^ and Mg^2+^	epirubicin injection solution, human urine samples	[38]
	GCE/Ag-MWCNT	SWV	0.003–0.25 μM, 1 nM	in 5000-folds of ascorbic acid, glucose; 2000-folds of uric acid, caffeine, vitamin A, vitamin E; 1000-folds of dopamine; 2000-folds of Na^+^, K^+^, Fe^2+^, Fe^3+^, Cu^2+^, Hg^2+^, Pb^2+^, Ca^2+^, Zn^2+^, Cl^−^, SO_4_^2−^, NO^3−^	blood, pharmaceuticals and urine samples	[39]
	SPCE/ERGO-PPFL_NADES_	EIS	1 pM–10 nM, 1 pM	in 10-folds of glucose, ascorbic acid, uric acid	artificial urine, human urine samples, medical preparation Idarubicin-Zavedos^®^	[This work]

Designations: GPE—graphite paste electrode, PC—perlite/cobalt oxide, PP—polypyrrole, NrG—nitrogen doped reduced graphene, SWCNT—single-walled carbon nanotubes, PGE—pencil graphite electrode, MWCNT—multiwalled carbon nanotubes, DES—deep eutectic solvent, CPE—carbon paste electrode, SPCE—screen-printed carbon electrode, GCE—glassy carbon electrode, GO—graphene oxide, rGO—reduced graphene oxide, MOF—metal–organic framework, mSiO_2_—alumina powder, MoS_2_—nanoflowers sodium molybdate dihydrate (Na_2_MoO_4_ H_2_O), NR—neutral red, PNR—poly(Neutral red), TKA—polycarboxylated thiacax[4]arene.

**Table 2:** Doxorubicin, epirubicin, and idarubicin determination in the presence of a 10-fold excess of glucose, ascorbic and uric acids by “added-found” approach. The average value ± S.D. for six independent sensors.

Sample	*R*_1_, kΩ	Recovery, %
1 µM doxorubicin (standard solution, *R*_1_ = 1.34 ± 0.05 kΩ)		
doxorubicin + glucose	1.31 ± 0.09	98 ± 7
doxorubicin + ascorbic acid	1.36 ± 0.09	102 ± 7
doxorubicin + uric acid	1.39 ± 0.08	104 ± 6
0.3 nM idarubicin (standard solution, *R*_1_ = 1.99 ± 0.20 kΩ)		
idarubicin + glucose	1.96 ± 0.22	98 ± 11
idarubicin + ascorbic acid	2.20 ± 0.13	110 ± 6
idarubicin + uric acid	1.65 ± 0.02	83 ± 1
0.1 nM epirubicin (standard solution, *R*_1_ = 1.40 ± 0.08 kΩ)		
epirubicin + glucose	1.31 ± 0.19	94 ± 14
epirubicin + ascorbic acid	1.38 ± 0.14	99 ± 10
epirubicin + uric acid	1.46 ± 0.07	104 ± 5

**Table 3:** Doxorubicin and idarubicin determination in pharmaceutical forms and standard solutions with stabilizers by “added-found” approach. The average value ± S.D. for six independent sensors.

Sample	*R*_1_, kΩ	Recovery, %
1 µM doxorubicin (standard solution, *R*_1_ = 1.34 ± 0.05 kΩ)		
Doxorubicin-TEVA^®^	1.28 ± 0.09	96 ± 7
Doxorubicin-LANS^®^	1.70 ± 0.09	127 ± 7
Doxorubicin with mannitol addition	1.53 ± 0.13	114 ± 10
0.3 nM idarubicin (standard solution, *R*_1_ = 1.99 ± 0.20 kΩ)		
Idarubicin-Zavedos^®^	1.60 ± 0.12	80 ± 6
Idarubicin with lactose addition	1.54 ± 0.13	77 ± 6

**Table 4:** Anthracycline determination in artificial urine and human urine samples by “added-found” approach. The average value ± S.D. for six independent sensors.

Sample	*R*_1_, kΩ	Recovery, %
1 µM doxorubicin (standard solution, *R*_1_ = 1.34 ± 0.05 kΩ)		
Human urine, non-diluted	1.88 ± 0.08	140 ± 6
Human urine, 1:1 diluted	1.44 ± 0.11	107 ± 8
Artificial urine, non-diluted	1.63 ± 0.07	122 ± 5
Artificial urine, 1:1 diluted	1.33 ± 0.11	99 ± 8
0.3 nM idarubicin (standard solution, *R*_1_ = 1.99 ± 0.20 kΩ)		
Human urine, non-diluted	1.97 ± 0.14	98 ± 7
Artificial urine, non-diluted	1.83 ± 0.14	93 ± 6
0.1 nM epirubicin (standard solution, *R*_1_ = 1.40 ± 0.08 kΩ)		
Human urine, non-diluted	1.89 ± 0.15	134 ± 6
Human urine, 1:1 diluted	1.36 ± 0.09	97 ± 10
Artificial urine, non-diluted	1.77 ± 0.09	126 ± 6
Artificial urine, 1:1 diluted	1.47 ± 0.06	104 ± 4

## Data Availability

The data presented in this study are available in the Supplementary Materials.

## Supplementary Materials

The following supporting information can be downloaded at https://www.mdpi.com/article/10.3390/bios15060385/s1, Figure S1. NADES and GO components mixture (a) after mixing with vortex and (b) after ultrasonication; Figure S2. Polyproflavine voltametric response stabilization after layer by layer (red) and “one-pot” (grey) modification of electrode surface; (a) oxidation peak currents, (b) reduction peak currents, 0.025 M PB, pH 7.0, potential range −0.6…0.6 V, 0.1 V/s; Figure S3. (a) GO electrochemical reduction towards ERGO, (b) potentiostatic and (c) potentiodynamic mode of proflavine electropolymerization; Figure S4. Cyclic voltammograms on SPCE modified with ERGO-PPFL_NADES_, 0.025 M PB with 0.1 M KCl, pH 7.0, −0.6…0.6 V, at scan rates 0.01, 0.04, 0.07, 0.1, 0.2, 0.3, 0.4 and 0.5 V/s; Figure S5. Dependence of EIS parameters on doxorubicin, epirubicin or idarubicin concentration: charge transfer resistance *R*_1_, (a,c,e) constant phase element *C_1_* (b,d,f); 0.025 M PB, pH = 7.0 in presence of 0.01 M [Fe(CN)_6_]^3−/4−^. Figure S6. Nyquist diagrams with average values and standard errors of -Z″ and Z′ for (a) doxorubicin, (b) epirubicin, (c) idarubicin. Layer content: 1—SPCE/ERGO-PPFL_NADES_, 2—SPCE/ERGO-PPFL_NADES_/DNAss, (3a)–(5a) SPCE/ERGO-PPFL_NADES_/DNAss/DOX (100 nM, 1 µM, 10 µM), (3b)–(5b) SPCE/ERGO-PPFL_NADES_/DNAss/EPI (10 pM, 100 pM, 1 nM), (3c)–(5c) SPCE/ERGO-PPFL_NADES_/DNAss/IDA (30 pM, 300 pM, 3 nM). Average value ± S.D. for six individual sensors. Control-SPCE/ERGO-PPFL_NADES_/DNA response; Table S1. Potential values for Nyquist diagrams recording. Table S2. Potential values for Nyquist diagrams recording.
