# Supplementary material for: Impedimetric DNA Sensor Based on a Composite of Electrochemically Reduced Graphene Oxide and Polyproflavine Electropolymerized from Natural Deep Eutectic Solvent for Anthracycline Medications Determination
Source: Biosensors (Basel). 2025 Jun 14;15(6):385. doi: 10.3390/bios15060385 (PMC12191363; doi:10.3390/bios15060385)
Supplement: Supplementary file 1 [file biosensors-15-00385-s001.zip › biosensors-3675911-supplementary.pdf]

Electronic Supporting Information

to the article by Anastasia Goida, Tatiana Krasnova, Rezeda Shamagsumova, Vladimir Evtugyn, Anatoly Saveliev, and Anna Porfireva

Impedimetric DNA Sensor Based on Composite of Electrochemically Reduced Graphene Oxide and Polyproflavine Electropolymerized from Natural Deep Eutectic Solvent for Anthracycline Medications Determination

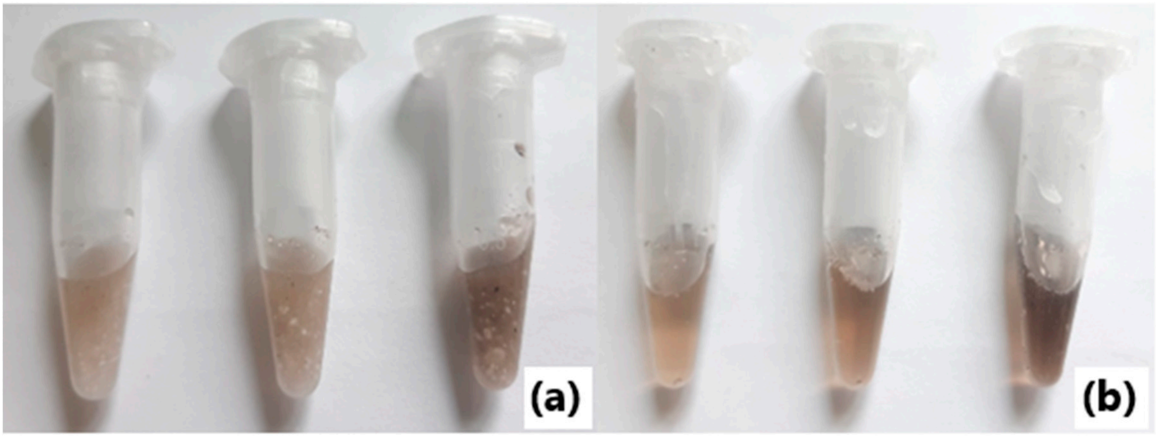

Figure S1 – NADES and GO components mixture (a) after mixing with vortex or (b) ultrasonication

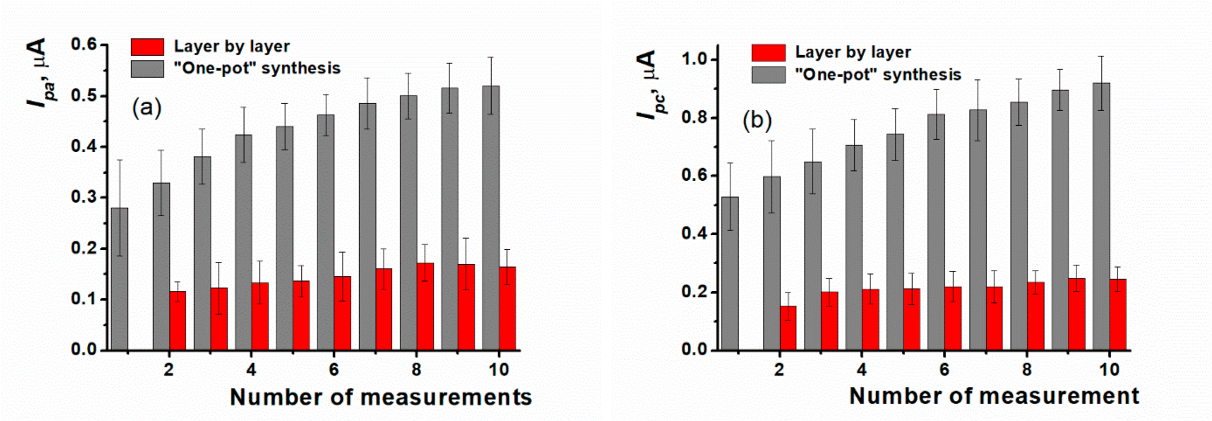

Figure S2 – Polyproflavine voltammetric response stabilization after layer by layer (red) and "one-pot" (grey) modification of electrode surface; (a) oxidation peak currents, (b) reduction peak currents, 0.025 M PB, pH 7.0, potential range -0.6…0.6 V, 0.1 V/s

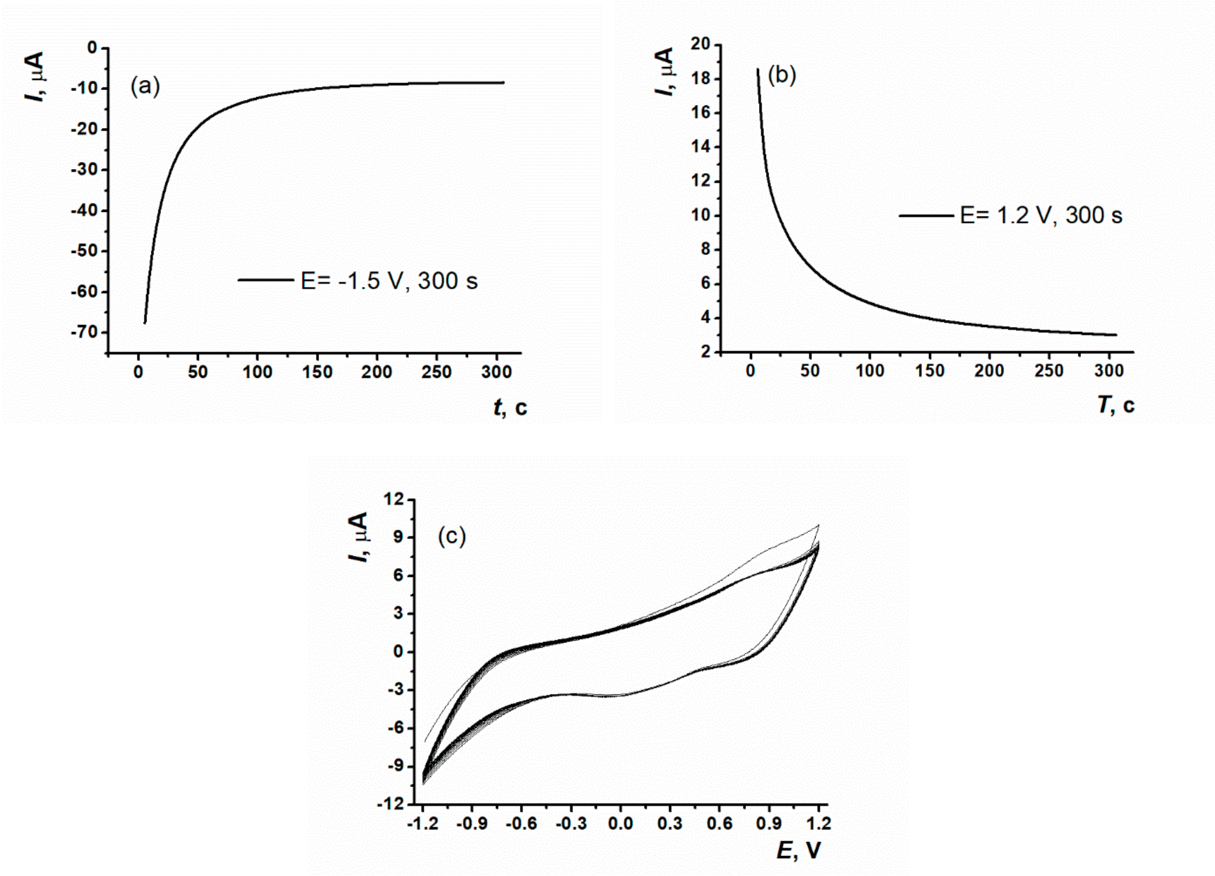

Figure S3 – a) GO electrochemical reduction towards ERGO, b) potentiostatic and c) potentiodynamic modes of proflavine electropolymerization

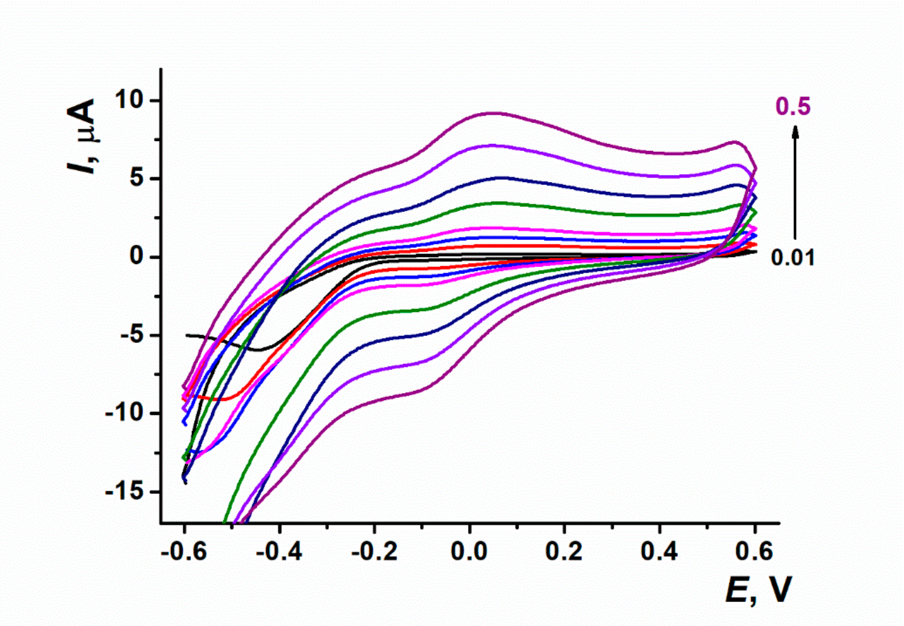

Figure S4 – Cyclic voltammograms on SPCE modified with ERGO-PPFL$_{NADES}$, 0.025 M PB with 0.1 M KCl, p□ 7.0, -0.6…0.6 V, at scan rates 0.01, 0.04, 0.07, 0.1, 0.2, 0.3, 0.4 or 0.5 V/s

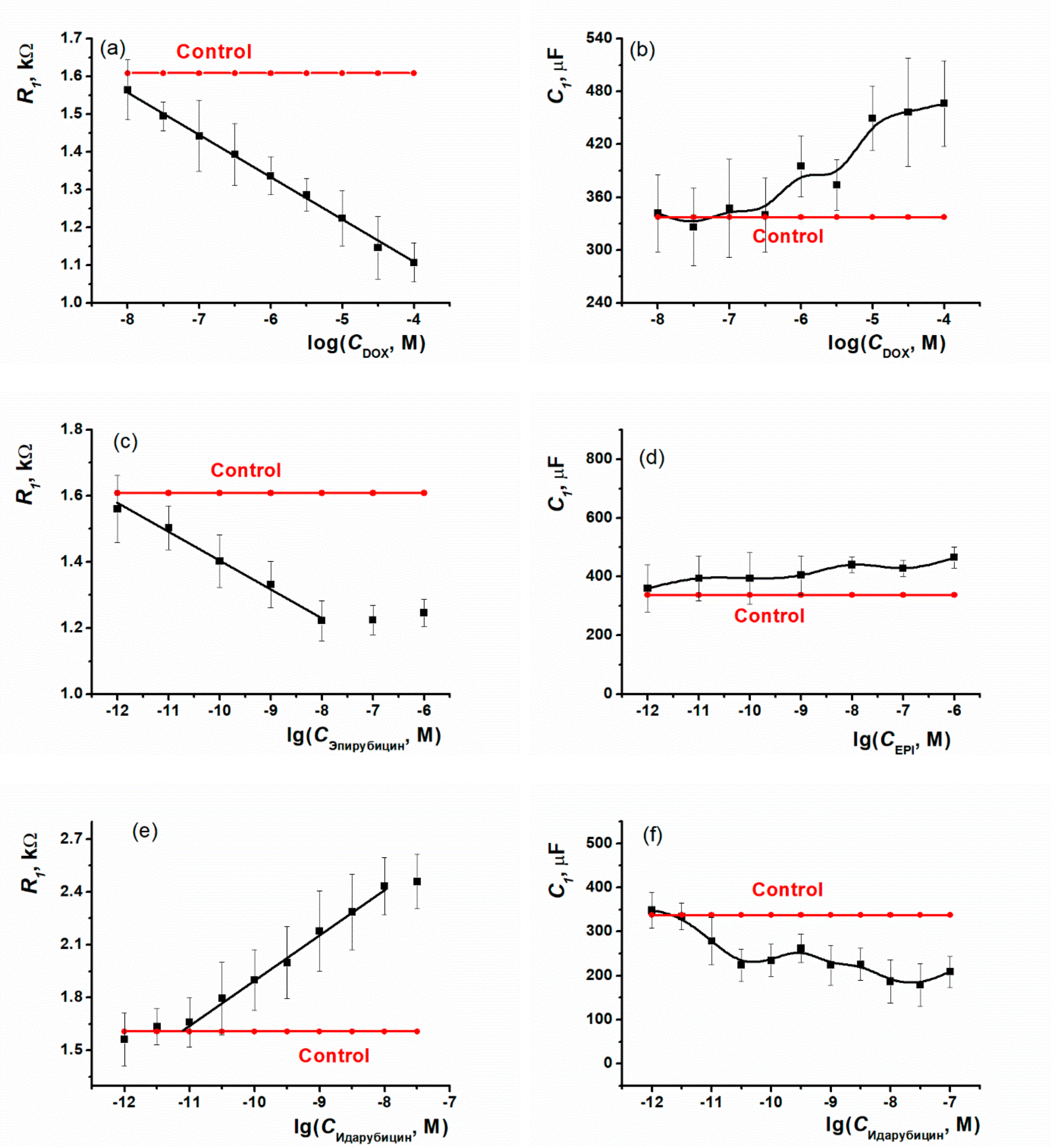

Figure S5 – EIS parameters dependency on doxorubicin, epirubicin or idarubicin concentration: charge transfer resistance R_1 (**a, c, e**), constant phase element C_1 (**b, d, f**); 0.025 M PB, pH=7.0 in presence of 0.01 M $[Fe(CN)_6]^{3-/4-}$. Average value ±S.D. for six individual sensors. Control – SPCE/ERGO-PPFL$_{NADES}$/DNA response

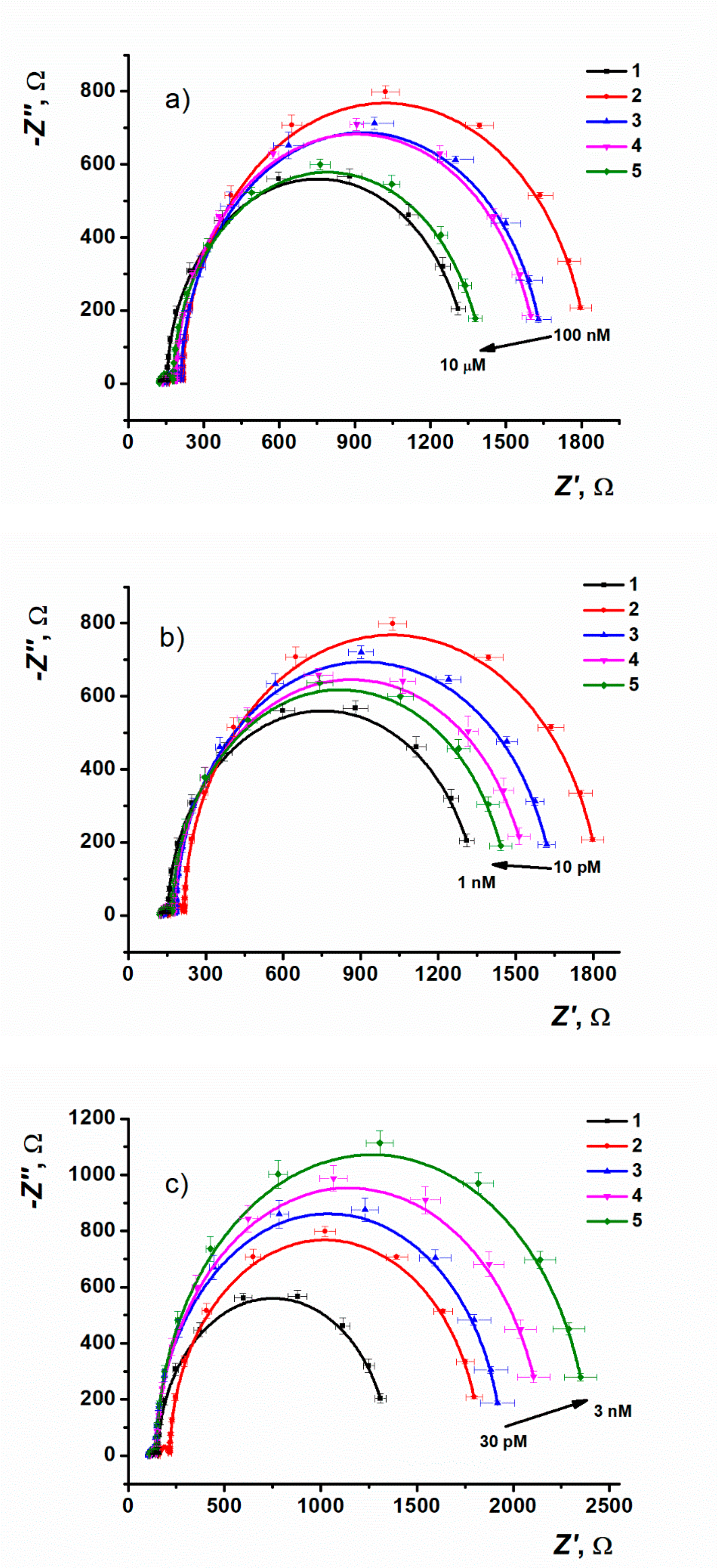

Figure S6 – Nyquist diagrams with average values and standard errors of -Z'' and Z' for (a) doxorubicin, (b) epirubicin, (c) idarubicin. Layer content: 1 – SPCE/ERGO-PPFL$_{NADES}$, 2 -

SPCE/ERGO-PPFL$_{NADES}$/DNAss, 3a)-5a) SPCE/ERGO-PPFL$_{NADES}$/DNAss/DOX (100 nM, 1 μM, 10 μM), 3b)-5b) SPCE/ERGO-PPFL$_{NADES}$/DNAss/EPI (10 pM, 100 pM, 1 nM), 3c)-5c) SPCE/ERGO-PPFL$_{NADES}$/DNAss/IDA (30 pM, 300 pM, 3 nM)

Table S1a – Results of elemental mapping data for SPCE/GO

Element	Line type	Weight %	Atomic %	Standard
C	K set	71.03	78.05	C Vit
O	K set	24.57	20.27	SiO2
Na	K set	0.05	0.03	Albite
Al	K set	0.03	0.02	Al2O3
Si	K set	0.26	0.12	SiO2
S	K set	0.20	0.08	FeS2
Cl	K set	3.85	1.43	NaCl
Total:		100.00	100.00	

Table S1b – Results of elemental mapping data for SPCE/ERGO$_{NADES}$

Element	Line type	Weight %	Atomic %	Standard
C	K set	90.72	95.04	C Vit
O	K set	3.79	2.98	SiO2
Si	K set	0.25	0.11	SiO2
S	K set	0.18	0.07	FeS2
Cl	K set	5.07	1.80	NaCl
Total:		100.00	100.00	

Table S1c – Results of elemental mapping data for SPCE/ERGO-PPFL$_{NADES}$

Element	Line type	Weight %	Atomic %	Standard
C	K set	89.86	94.93	C Vit
N	K set	0.00	0.00	BN
O	K set	3.22	2.56	SiO2
Al	K set	0.05	0.03	Al2O3
Si	K set	0.30	0.14	SiO2

S	K set	0.20	0.08	FeS2
Cl	K set	6.35	2.27	NaCl
Total:		100.00	100.00	

Table S2 – Potential values for Nyquist diagrams recording

Layer content	E_m		
SPCE	0.117 V		
SPCE/PPFL	0.115 V		
SPCE/PPFL/DNA	0.145 V		
	Epirubicin	Doxorubicin	Idarubicin
SPCE/PPFL/medication	0.116 V	0.133 V	0.114 V
